# Photoelectron-Photofragment Coincidence Spectroscopy With Ions Prepared in a Cryogenic Octopole Accumulation Trap: Collisional Excitation and Buffer Gas Cooling

**DOI:** 10.3389/fchem.2019.00295

**Published:** 2019-04-30

**Authors:** Ben B. Shen, Katharine G. Lunny, Yanice Benitez, Robert E. Continetti

**Affiliations:** Department of Chemistry and Biochemistry, University of California, San Diego, San Diego, CA, United States

**Keywords:** ion trap, ozonide, photoelectron, photofragment, coincidence spectroscopy, collisional excitation, buffer gas cooling, ozone

## Abstract

A cryogenic octopole accumulation trap (COAT) has been coupled to a photoelectron-photofragment coincidence (PPC) spectrometer allowing for improved control over anion vibrational excitation. The anions are heated and cooled via collisions with buffer gas <17 K. Shorter trapping times (500 μs) prevent thermalization and result in anions with high internal excitation while longer trapping times (80 ms) at cryogenic temperatures thermalize the ions to the temperature of the buffer gas. The capabilities of the COAT are demonstrated using PPC spectroscopy of ${\text{O}}_{3}^{-}$ at 388 nm (E_hν_ = 3.20 eV). Cooling the precursor anions with COAT resulted in the elimination of the autodetachment of vibrationally excited ${\text{O}}_{2}^{-}$ produced by the photodissociation ${\text{O}}_{3}^{-}$ + hν → O + ${\text{O}}_{2}^{-}$(v ≥ 4). Under heating conditions, a lower limit temperature for the anions was determined to be 1,500 K through Franck-Condon simulations of the photodetachment spectrum of ${\text{O}}_{3}^{-}$, considering a significant fraction of the ions undergo photodissociation in competition with photodetachment. The ability to cool or heat ions by varying ion injection and trapping duration in COAT provides a new flexibility for studying the spectroscopy of cold ions as well as thermally activated processes.

## Introduction

Measurements of energy partitioning for neutral dissociation processes provide significant insights into the chemistry of transient species, providing benchmarks for understanding reaction dynamics (Johnson et al., 2014; Otto et al., 2014a). Experimentally, such measurements are challenging due to the complexity of potential energy surfaces and require spectroscopic probes for all resulting products of a photo-induced process. Anion photodetachment coupled with photoelectron-photofragment coincidence (PPC) spectroscopy provides a broad overview of the reaction dynamics at a fixed photon energy. The photoelectron kinetic energy (eKE) spectrum encodes the distribution of potential energies on the nascent neutral surface, and the corresponding eKE-resolved kinetic energy release (KER) spectra for the neutral products provides a measure of the subsequent dissociation mechanism. In addition, the ability to distinguish photodetachment processes that yield stable neutral products as opposed to dissociative photodetachment (DPD) provides valuable information. However, internal excitation in the precursor anions yields congested PPC spectra (Corderman and Lineberger, 1979; Hock et al., 2012; Boyarkin and Kopysov, 2014; Johnson et al., 2014) leaving ambiguity in the energy available to the neutral fragments in DPD processes. Preparation of precursor anions with known internal energies greatly enhances the ability to determine the energy partitioning for the dissociative pathways. In an effort to better control the internal excitation of precursor anions, a cryogenic octopole accumulation trap (COAT) has been coupled to an existing photoelectron-photofragment coincidence (PPC) spectrometer enabling the preparation of both hot and cold precursor anions as demonstrated through PPC spectroscopy of ${\text{O}}_{3}^{-}$.

Spectroscopic studies of ions with high internal excitation yields complex and congested spectra, often obscuring features that yield information about reaction dynamics. Supersonic expansions have been broadly applied in gas-phase experiments for producing molecules with vibrational temperatures below 100 K and rotational temperatures below 20 K (Smalley et al., 1977). Unfortunately, the ionization process itself can induce significant amounts of internal energy, resulting in system-dependent cooling efficacy (Sanz et al., 2005; Johnson et al., 2014; Shen et al., 2014). Previously in PPC experiments, progress was made in the preparation of colder ions by implementing an electrostatic ion beam trap (EIBT). This decoupled the kHz PPC measurements in the EIBT from the pulsed ion source, allowing for a low-repetition rate (10 Hz) for more effective cooling in stronger supersonic expansions. This was demonstrated on small molecules such as HOCO^−^, giving a detailed characterization of deep tunneling involved in the dissociation of *cis*-HOCO to H + CO_2_ (Johnson et al., 2011). Unfortunately, larger ions such as *tert*-butoxide ((CH_3_)_3_CO^−^) are not efficiently cooled in a supersonic expansion. This results in observed dissociation dynamics only accessible through non-Boltzmann population of highly vibrationally excited anions (Shen et al., 2014). Results like these have motivated the search for improved methods for producing cold anions in kHz repetition-rate PPC experiments.

Various methods of preparing cold anions were considered for the PPC spectrometer including buffer-gas-cooled radiofrequency (RF) ion traps. Due to the low-duty cycle, a RF ion trap alone is not an ideal approach to performing PPC measurements. PPC measurements provide kinematically complete information on events that lead to a free electron and momentum-matched neutral products detected in coincidence, so a high duty cycle and low event rate are required to minimize contamination from false coincidences (Continetti, 1998, 2001; Stert et al., 1999). Addition of the EIBT to the PPC spectrometer decoupled the source duty cycle from the photodetachment laser interaction duty cycle through the recycling of ions within the EIBT. This allowed the source repetition rate to be reduced to 10 Hz, while maintaining the high PPC data acquisition duty cycle (1 kHz) required to carry out successful multi-particle coincidence experiments. The decoupling of the ion source and EIBT duty cycles paved the way for application of a low-repetition-rate cryogenic radiofrequency (RF) trap as a method for cooling ions prior to PPC measurements.

RF ion traps have been proven to be a robust method of storing and cooling ions via collisions with cold buffer gas and have been extensively used in cooling clusters and larger biomolecules (Gerlich, 1995; Jasik et al., 2013; Redwine et al., 2013; Boyarkin and Kopysov, 2014). The cooling ability of the RF trap is limited not only by the temperature of the ion trap, but also by ion heating induced by the RF electric field (Gerlich, 1995). Within a RF trap, ion oscillation at the frequency of the RF field causes heating through collisions with the buffer gas. The oscillation is highly dependent on the effective potential (V_eff_) within the trap. Quadrupole traps have a parabolic effective potential, and the large field-induced gradient is expected to lead to a propensity for significant RF heating. Increasing the order (number of electrodes) for a linear RF trap creates an effective potential with a smaller radial field gradient through the flattening of the minima. Gerlich et al. demonstrated with a 22-pole trap that this creates a steep rise in potential near the RF rods (Gerlich, 1995). This allows for a larger area in the center of the trap relatively free of RF heating within which the ions are confined. In the present application, RF ion traps provide flexibility in the preparation of ions for PPC spectroscopy by allowing for cooling as well as heating of ions. The production of cold anions allows for more clearly defined ion energetics for the subsequent study of photo-induced processes. Alternatively, the ability to collisionally heat the ions allows investigation of the effects of internal excitation on dissociation dynamics and thermally activated processes.

**Figure 1:**
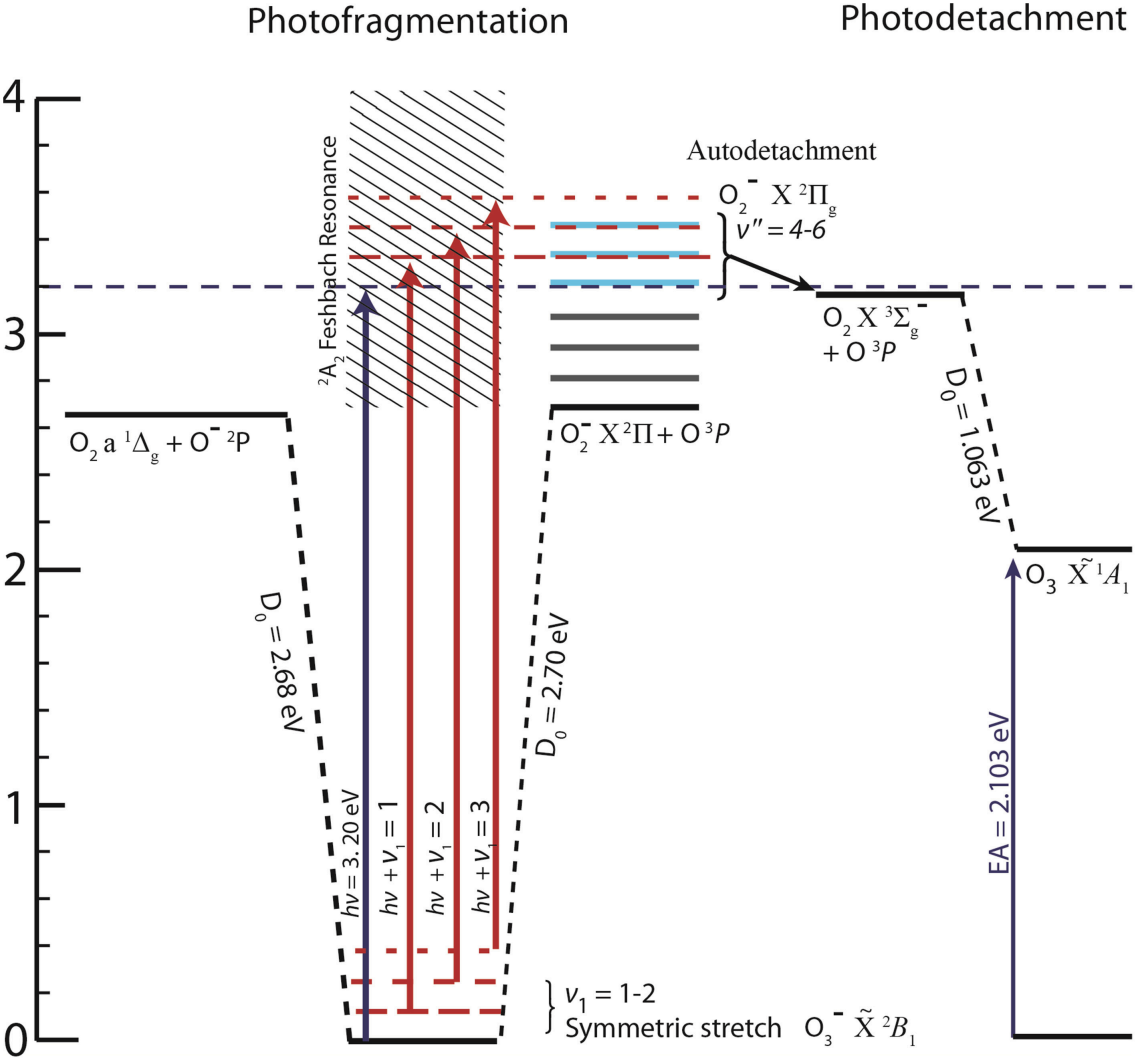
The energetics of available pathways relative to ground state ${\text{O}}_{3}^{-}$ following photoabsorption at E_hν_ = 3.20 eV (Novick et al., 1979; Arnold et al., 1994), including the Feshbach resonance identified in DEA studies (Allan et al., 1996a; Rangwala et al., 1999), O_2_ energetics (Ervin et al., 2003), and relevant excited states of ${\text{O}}_{3}^{-}$ (Cui and Morokuma, 1998). Dashed horizontal red lines indicate internal excitation in the anion resulting in opening the otherwise energetically inaccessible autodetachment channel.

To demonstrate the effectiveness of COAT, PPC spectroscopy was performed on ${\text{O}}_{3}^{-}$ at 388 nm (E_hν_ = 3.20 eV), just below the threshold for autodetachment of ${\text{O}}_{2}^{-}$(v ≥ 4) products which result from the photodissociation of ${\text{O}}_{3}^{-}$. The photoelectron spectrum of ${\text{O}}_{3}^{-}$ at E_hν_ = 3.20 eV show three concurrent photophysical processes: (1) photodetachment O_3_– + hν → O_3_ + e^−^; (2) photodissociation/autodetachment ${\text{O}}_{3}^{-}$ + hν → ${\text{O}}_{2}^{-}$(^2^Π) + O(^3^P), and (3) photodissociation ${\text{O}}_{3}^{-}$ + hν → O^−^(^2^P) + O_2_(${}^{1}\Delta_{\text{g}}$) followed by the photodetachment O^−^(^2^P) + hν → O(^3^P) by a second photon. While all three processes are influenced by the internal excitation of the precursor anion, the most striking effect is observed in process (2). It is well-known that vibrational excitation of ${\text{O}}_{2}^{-}$ > 4 quanta results in autodetachment of the electron yielding O_2_(${}^{3}\Sigma_{\text{g}}^{-}$) (Allan et al., 1996a; Matejcik et al., 1996; Goebbert and Sanov, 2009). Jarrold et al. recently observed the autodetachment of ${\text{O}}_{2}^{-}$ as a result of photofragmentation of ${\text{O}}_{3}^{-}$ to ${\text{O}}_{2}^{-}$ (v > 4) + O at 355 nm (E_hν_ = 3.49 eV) photon energy (Nestmann et al., 2005), and this process was further characterized at E_hν_ = 3.20 eV in an initial report from our laboratory (Shen et al., 2017). The onset for the autodetachment channel lies 3.24 eV above ${\text{O}}_{3}^{-}$(${\tilde{X}}^{2}$B_1_) as shown in Figure 1. Access to the autodetachment channel is therefore energetically accessible only when the precursor anion (${\text{O}}_{3}^{-}$) is vibrationally excited, making this an ideal candidate for testing the capabilities of COAT. The design and application of COAT in PPC spectroscopy is discussed in detail in the following sections, using ${\text{O}}_{3}^{-}$ as an example of the ability to prepare either cold or hot ions to exert control over product channel pathways.

## Experimental Setup

**Figure 2:**
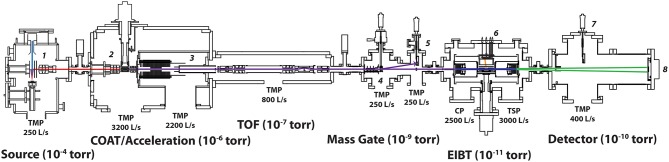
Overview of the modified PPC spectrometer incorporating a new source and COAT. Labeled sections are as follows: (1) Source with pulsed valve/discharge assembly with Wiley-McLaren-style extraction. (2) COAT (3) Acceleration stack with potential switch. (4) Electrostatic chopper. (5) Pre-EIBT ion detector. (6) Electron Detector / EIBT. (7) Post-EIBT ion detector. (8) Neutral particle detector.

The cryo-PPC spectrometer (Johnson et al., 2011) has been modified to include a new source chamber and a cryogenic octopole accumulation trap (COAT) as shown in Figure 2. The new modifications can be divided into three sections discussed in detail below: ion source, COAT trap design, and COAT operation.

### Ion Source

A new source chamber was added to the existing PPC spectrometer to house a piezoelectric pulsed valve (PPV) with coaxial discharge plates and a Wiley-McLaren style mass spectrometer. This source chamber is pumped by an Edwards NEXT240 turbomolecular pump maintaining 10^−4^ mbar pressure during operation. Ions are generated in a supersonic expansion from the PPV with a 1 keV electron beam counter-propagating down the expansion, oriented perpendicular to the ion beam axis of the PPC spectrometer. The ions are extracted from the expansion using three pulsed electrodes in a Wiley-McLaren configuration (Wiley and McLaren, 1955). A gear system was constructed to allow the distance between the PPV and the Wiley-McLaren plates to be adjusted, enabling extraction of different portions of the supersonic expansion. The first two plates (14 cm outside diameter with 2 cm inner diameter apertures) are spaced 6 cm apart between which the supersonic expansion propagates. The plates are pulsed with negative potentials giving ions an average of ~225 eV translational kinetic energy while the third plate is typically held at −30 V. The ions are then guided through six focusing lens elements and one set of deflectors into COAT in the next chamber.

### Coat Trap Design

**Figure 3:**
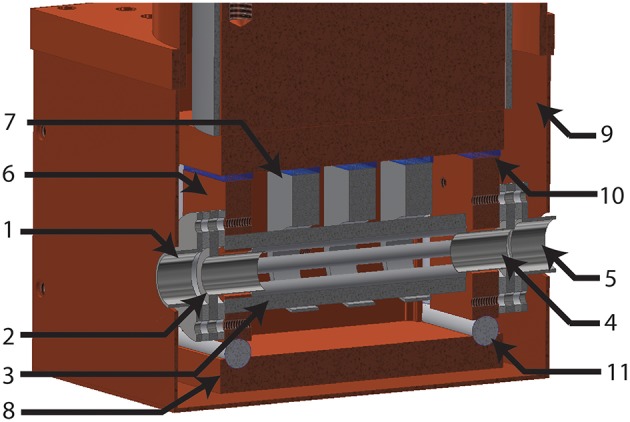
Cross sectional view of the COAT assembly. Labeled components are as follows: (1) Entrance lens. (2) Entrance endcap. (3) RF rods. (4) Exit endcap. (5) Exit lens. (6) RF rod mount. (7) Shaping electrodes. (8) Buffer gas shield. (9) Thermal radiation shield. (10) Sapphire insulation. (11) Macor spacers.

COAT is a linear octopole trap, as shown in Figure 3, based on a similar design used by Wester et al. (Otto et al., 2012). These devices have been known to be effective at cooling both external and internal degrees of freedom via buffer gas collisions (Gerlich, 1992; Wester, 2009; Otto et al., 2012; Redwine et al., 2013). For linear RF traps, the effective radial field is V_eff_(r) ∞ r^n−2^, where r is the radius and n is the number of poles (Gerlich, 1992, 1995). The V_eff_ plays a critical role in determining the trapping volume, as mentioned previously. An octopole configuration provides an optimal compromise in trapping volume by confining the ions along a smaller radius from the center of the trap, while still allowing for facile extraction of the ions. This also allows for maximal laser overlap with the trapped ions for future IR excitation experiments.

The octopole trap is composed of RF rods with a 2.5 mm diameter arranged in a cylindrical array, with an inscribed inner diameter of 7.5 mm. The assembly (Figure 3) consists of four cylindrical rods mounted to each RF mount and assembled such that adjacent rods are mounted on opposing rod mounts. The trapping of ions in the radial direction is achieved by applying opposite RF phase to each RF rod mount giving adjacent cylindrical rods an alternating RF phase, generating an octopole field with a 4 MHz, 320 volt peak-to-peak RF waveform produced by a home-built RF generator (Jones et al., 1997; Jones and Anderson, 2000). Longitudinal confinement is achieved by endcaps on both sides of COAT with a 6 mm diameter opening which can be switched, using home built high voltage MOSFET switches, for loading, trapping, and extracting ions. Three equally spaced shaping electrodes surround the RF rods and, with limited field penetration, generate a potential field ramp to bias longitudinal storage of ions toward the exit endcap of the trap. The RF mounts, as well as the shaping electrodes, are all electrically isolated from the copper base of the trap by sapphire plates, taking advantage of sapphire's high thermal conductivity at cryogenic temperatures. The RF mounts along with a buffer gas shield surrounds the rods and shaping electrodes to provide a cold closed environment for collisional cooling. A thin layer of Apiezon N is used between all areas of mechanical contact to improve thermal conductivity at cryogenic temperatures.

The entire trap is mounted on a heating block that allows for variable temperatures between ~10 and 300 K via heaters clamped around the heating block. The heating block, in turn, is mounted to the 2nd stage of a Sumitomo RDK-205D 4K Cryocooler cold head. COAT can be cooled to ~17 K as measured by a silicon diode (LakeShore DT-471-CO) though it is an upper limit as H_2_ freezes onto the electrodes of COAT indicating an inner surface temperature of ~10 K. The 1st stage of the cold head is mounted to a thermal radiation shield (37 K) that encloses COAT. The buffer gas is pre-cooled to ~40 K through a 3 mm diameter copper tube in thermal contact with the 1st stage of the cold head prior to injection into COAT through a hole in the base of the trap. The entire assembly is mounted on a movable flange on top of the COAT chamber allowing for the COAT to be aligned or moved out of the ion beam-line axis.

### Coat Operation

COAT is generally operated in one of three modes: cooling, heating or accumulation. The ability to heat and cool ions comes from controlling the initial translational energy of the precursor ions as they enter into COAT, as well as adjusting the duration of trapping. To reduce the initial translational energy of the ions entering the trap, the entire trap assembly is floated at an appropriate DC potential, and the incoming ions are focused into the trap using an entrance lens element. To further facilitate trapping and cooling of the ions, a pulse of pre-cooled buffer gas generated with a Gerlich-type valve (Gerlich et al., 2019) is used to raise the pressure in the trap to ~10^−2^ mbar prior to ion injection. Upon entering, the ions collide with buffer gas, further reducing translational kinetic energy, and trapping them within COAT.

**Figure 4:**
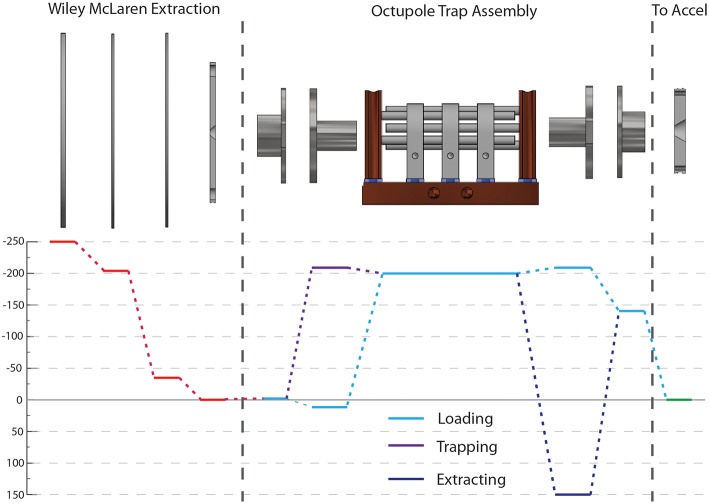
A voltage map for the new source is shown indicating typical voltages ions experience (not to scale). The red lines indicate the voltages for the Wiley McLaren extraction. Within COAT, there are three distinct stages of performance: ion loading (light blue), trapping (purple), and extraction (dark blue). The green line indicates the voltage of the entrance to the acceleration stage.

All source timing signals are controlled by a Stanford Research DG645 digital delay generator triggered by and prescaled to 1/100th of the laser repetition rate (1,037 Hz) while all COAT timings are controlled by a Quantum Composer 9,518 digital delay generator triggered off the prescaled signal. These timings control what mode COAT is run in: accumulation, cooling, or heating. In accumulation mode, the entrance endcap can be held at constant trapping voltage to facilitate accumulation of ions over multiple ion generation cycles. This can be synchronized with a faster rate of ion generation (20 Hz) and/or coupled with a longer EIBT trap time. In accumulation mode, only cold ions are available since most of the ions are trapped for a long period. In cooling and heating mode, the entrance endcap is switched from an attractive potential while loading ions to a repulsive potential to trap ions. This maximizes the quantity of ions entering the trap for a single cycle. A typical map of voltages on the essential elements is shown in Figure 4 for the cooling/heating mode.

Within COAT, the ions undergo collisional cooling with the buffer gas for a pre-set period of time determined by whether or not vibrationally excited ions are desired. Shorter trapping time limits the thermalization and cooling of the ions, allowing the preparation of hotter ions. Increasing the trapping time allows thermalization of the trapped ions to the temperature of COAT. Similar traps have shown thermalization of the anions under similar pressures (~10^−2^ mbar) within 30 ms (Hock et al., 2012). Due to coupling with our EIBT, a trapping time of 80 ms is typical for maximizing the cooling time while still maintaining a 10 Hz duty cycle. After the set trapping time, the exit endcap is switched to an attractive potential to extract the ions from within COAT, and the ions guided into the next portion of the apparatus with three focusing elements.

The ions are then directed into a differentially pumped chamber where they are accelerated to a kinetic energy of 7 keV, re-referenced to ground (Figure 2, region 3), and mass selected by time of flight (Figure 2, region 4) for trapping within a cryogenically cooled electrostatic ion beam trap (EIBT) for 100 ms (Figure 2, region 6). This aspect of the experiment has been described in detail previously (Johnson et al., 2011). Within the EIBT, the ion packet is bunched and phase-locked to a 387.8 nm (E_hν_ = 3.20 eV) laser pulse from a Ti:Sapphire regenerative amplifier (Clark MXR CPA-2000; 1.2 ps pulse width) at a repetition rate of 1,037 Hz using a field-programmable-gate-array-synced RF function generator (HP 3325). The oscillating ion packet interacts with the laser repeatedly over a 100 ms trapping period per experimental cycle, and the electron and neutral products are measured. The laser fluence is modulated by using a single 0.5 m focal length lens for high power density measurements vs. collimation with a 2.5:1 telescope on the 3.5 mm-diameter doubled output beam of the Ti:Sapphire laser. The power density for the collimated laser was estimated to be 2 × 10^9^ W/cm^2^ with the focused laser approximately 100x greater. Detached electrons are orthogonally extracted and mapped via velocity map imaging to a time and position sensitive detector. The center-of-mass eKE is determined from the three-dimensional electron velocity vector. Optimal resolution is achieved through selection of electrons with minimal z-velocities perpendicular to the detection plane as determined by the TOF of the center-of-mass for photoelectron detection by equatorially slicing the photoelectron spectra. This effect of slicing on the intensities in the photoelectron spectra was corrected for by dividing the sliced photoelectron spectrum by the energy-dependent acceptance function of the z-velocity slice (Bowen and Continetti, 2004). Calibration of ${\text{O}}_{2}^{-}$ photoelectron spectra as well as the O^−^ 2-photon events observed in the present experiments indicate ΔeKE/eKE ~4% full-width-at-half-maximum (FWHM) at 1.74 eV for O^−^. After photodetachment, the resulting neutrals, no longer trapped within the EIBT, exit and impinge on a multiparticle time-and position-sensitive detector 1.3 m away from the laser interaction region (region 8), allowing determination of the product mass ratio and kinetic energy release (KER) for each event.

## Results

**Figure 5:**
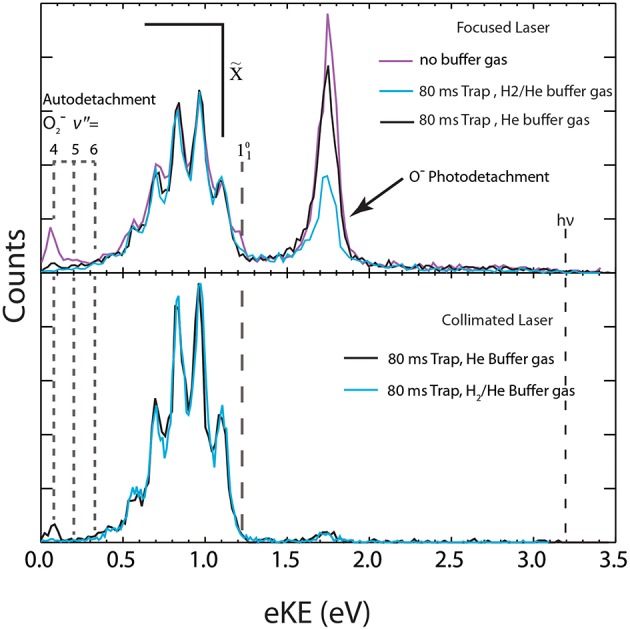
The total photoelectron spectrum for ${\text{O}}_{3}^{-}$ with different buffer gas and laser configurations is shown. Top panel (high laser fluence): spectra collected without buffer gas (purple trace), with H_2_/He buffer gas (blue trace) and with neat He buffer gas (black trace). Bottom panel (low laser fluence): Both ${\text{O}}_{3}^{-}$ spectra collected at 17 K with 80 ms trapping time with either neat He buffer gas (black trace) or H_2_/He buffer gas (blue trace).

**Figure 6:**
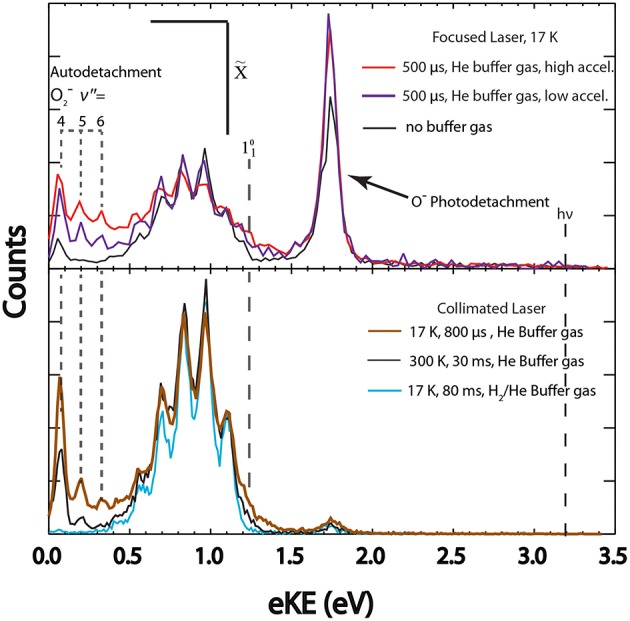
The total photoelectron spectrum for ${\text{O}}_{3}^{-}$ with different temperatures, trap times, and laser configurations is shown. Top panel (high laser fluence): Spectra collected with short trapping times (500 μs) with higher (red trace) and lower (purple trace) entrance lens acceleration compared to no buffer gas (black trace). Bottom panel (low laser fluence): Short trapping time at cold temperatures (800 μs, 17 K, neat He buffer gas) shown in brown, longer trapping times at room temperature (30 ms, 300 K, neat He buffer gas) shown in black, long trapping times at cold temperatures (80 ms, 17 K, H_2_/He buffer gas) shown in blue.

The capabilities of COAT are demonstrated here by comparing the signatures of the three concurrent photophysical channels occurring at E_hν_ = 3.20 eV in the photoelectron spectra of ${\text{O}}_{3}^{-}$: (1) photodetachment ${\text{O}}_{3}^{-}$ + hν → O_3_ + e^−^, (2) photodissociation/autodetachment ${\text{O}}_{3}^{-}$ + hν → ${\text{O}}_{2}^{-}$(${}^{2}\Pi_{\text{g}}$) + O(^3^P), and (3) photodissociation ${\text{O}}_{3}^{-}$ + hν → O^−^(^2^P) + O_2_(${}^{1}\Delta_{\text{g}}$) followed by the photodetachment O^−^(^2^P) + hν → O(^3^P) by a second photon. The primary spectroscopic feature of channel (1) is a structured photoelectron spectrum in the 0.30–1.50 eV eKE range (Novick et al., 1979; Arnold et al., 1994; Garner et al., 1997; Mann et al., 2015). Channel (2) is observed in the spectra as a structured signal at low eKE (0.0–0.4 eV) originating from sequential autodetachment of ${\text{O}}_{2}^{-}$(v″ > 4). In addition, at high laser fluence there is also a 2-photon ${\text{O}}_{2}^{-}$(v″ < 4) signal, observed as a broad baseline extending out to near the photon energy, produced by channel (2). In the second ion photodissociation channel (3), the photodissociation of ${\text{O}}_{3}^{-}$ results in stable O^−^, which is then photodetached at high laser fluence and results in a peak at eKE = 1.74 eV. The effective laser fluence was changed in order to distinguish one and two photon processes. The features from these three channels exhibit varying intensities with different COAT temperatures, trapping times, and buffer gas identity. These features will be compared and interpreted with a temperature estimate for the photodetachment channel using a Franck-Condon simulation. Under no buffer gas conditions, as seen in the upper panel of Figure 5 (purple trace) and the upper panel of Figure 6 (black trace) no collisional heating or cooling takes place, which will be used as a reference for the initial internal excitation of the trapped anions.

### Cooling

Over the course of an ion trapping cycle, ions within COAT collide with the pre-cooled buffer gas to remove both translational kinetic energy and internal energy, making the cooling performance dependent on buffer gas density and identity. The effects of varying buffer gas conditions can be seen in the total photoelectron spectra (electrons in coincidence with both dissociative products and stable O_3_) in Figure 5. The peaks at low eKE (0.07, 0.19, and 0.32 eV) originate from the photodissociation/autodetachment ${\text{O}}_{3}^{-}$ + hν → ${\text{O}}_{2}^{-}$(^2^Π) + O(^3^P) pathway (2) via photoexcitation to the ^2^A_2_ excited state as previously reported (Mann et al., 2015; Shen et al., 2017). At E_hν_ = 3.20 eV, access to ${\text{O}}_{2}^{-}$(v″ = 4,5,6) is energetically forbidden without vibrational excitation of ${\text{O}}_{3}^{-}$, as shown in the energetics diagram in Figure 1, so observation of vibrationally auto detaching ${\text{O}}_{2}^{-}$ provides a sensitive test of parent anion internal energy. The peaks at eKE = 0.07 and 0.19 eV correspond to the vibrational autodetachment of O_2_(v′ = 0) + e^−^ ← ${\text{O}}_{2}^{-}$(v″ = 4,5) and are most prominent under no buffer gas conditions, indicating significant initial vibrational excitation in ${\text{O}}_{3}^{-}$.

Trapping the ions for 80 ms in COAT at a temperature <17 K significantly reduces the autodetachment channel when neat He (black trace) buffer gas was used and the channel is effectively eliminated when a 20:80 H_2_/He (blue trace) buffer gas mix was used. The temperatures noted are the measured temperatures of COAT, but their relation to the ion temperature is dependent on the duration of trapping to allow for thermalization. Even with longer trapping times there may be non-Boltzmann distributions of excitation in high-frequency vibrations. The 20:80 H_2_/He buffer gas mix cools the precursor ions more effectively than neat He due to collisional cooling being more effective with lighter buffer gases (Moriwaki et al., 1992, 1996). The empirical efficacy of He/H_2_ gas mixtures for collisional cooling was first reported by Wang et al. in photoelectron spectroscopy studies (Wang and Wang, 2008), and has also been found to be effective in other systems (Kamrath et al., 2011; Hock et al., 2012), including ${\text{O}}_{3}^{-}$ as shown here.

The feature at 1.74 eV eKE is a result of (3) photodissociation ${\text{O}}_{3}^{-}$ + hν → O^−^(^2^P) + O_2_(${}^{1}\Delta_{\text{g}}$) followed by sequential photodetachment O^−^(^2^P) + hν → O(^3^P) (Shen et al., 2017). As shown in the upper panel (Figure 5), using a focused laser the O^−^ photodetachment signal is much stronger, while use of a collimated laser caused a significant decrease in this two photon signal, increasing the sensitivity to the hot bands in the stable channel. Upon cooling (Figure 5, upper panel, black line), the fraction of events resulting in channel (3) is reduced compared to the no-buffer-gas conditions (purple line) under similar laser fluence. The reduction in the signal under the H_2_/He condition is due to lower laser fluence rather than an effect of cooling.

**Figure 7:**
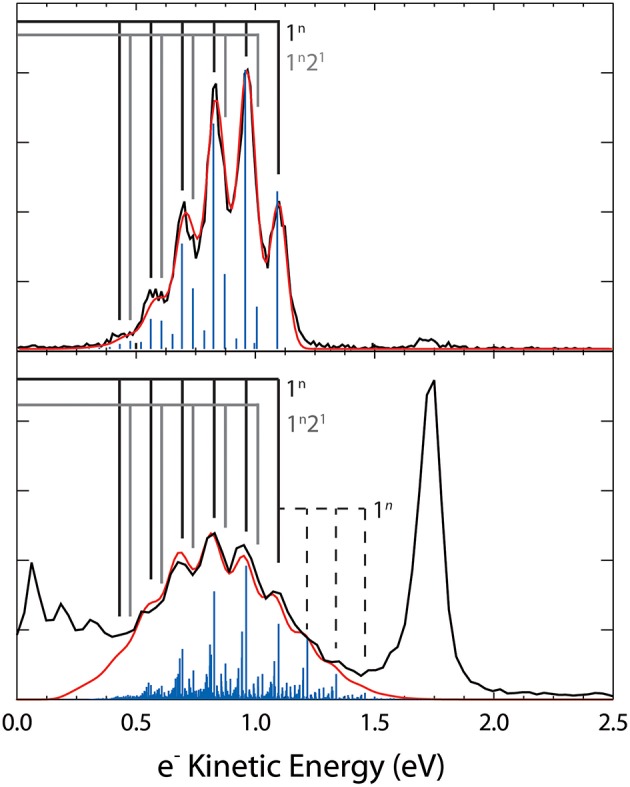
Top panel: Coldest ${\text{O}}_{3}^{-}$ spectrum, shown in black (80 ms Trap H_2_/He buffer gas) with Franck-Condon simulation (blue trace) assuming a 0 K ion temperature. Bottom panel: hottest ${\text{O}}_{3}^{-}$ spectrum, shown in black (500 μs, He buffer gas, high accel) with Franck-Condon simulations assuming *v*_1_ = 2,000 K and *v*_2_ = 1,500 K. The vibrational combs indicate the dominant transitions with the dashed comb indicating hot bands. Both simulations were Gaussian convoluted to generate the simulated spectra, shown in red.

The dominant channel observed in the total photoelectron spectra is the photodetachment (1) ${\text{O}}_{3}^{-}$ + hν → O_3_ + e^−^ yielding a structured photoelectron spectrum in the eKE range between 0.30 and 1.50 eV as shown in Figure 5. The photoelectron spectra are consistent with the electron affinity (EA) of O_3_ previously determined to be 2.10 eV and a Franck-Condon vibrational progression in the totally symmetric *v*_1_ and *v*_2_ modes of the O_3_(${\tilde{X}}^{1}$A_1_) ground state (Arnold et al., 1994). The vibrational energies for ${\text{O}}_{3}^{-}$ have been previously reported (Arnold et al., 1994) and are summarized in Table 1 with the $1_{1}^{0}$ hot band location annotated in Figure 5. Under the coldest conditions, the $1_{1}^{0}$ hot band is within the noise of the spectra indicating little if any population in the ν_1_ mode of ${\text{O}}_{3}^{-}$. Franck-Condon simulations (Figure 7) for the stable (1) ${\text{O}}_{3}^{-}$ + hν → O_3_ + e^−^ channel have been carried out with PESCAL (Ervin et al., 1988) using single point calculations in Gaussian 03 (Frisch et al., 2004) with previously reported geometries (Arnold et al., 1994; Liang et al., 2007) and frequencies (Table 1) (Arnold et al., 1994). The Franck-Condon factors were calculated using the independent Morse oscillator approximation due to the strong effects of anharmonicity for transitions to high vibrational levels of O_3_. The simulated spectra were generated by convolving the stick spectra with a Gaussian convolution consistent with the 4% ΔeKE/eKE resolution. The stick spectra were calculated at 0 K in all modes for cold conditions (top panel, ν_1_, ν_2_, ν_3_ = 0 K) and a Boltzmann distribution for hot conditions (bottom panel, ν_1_ = 2,000 K ν_2_ = 1,500 K ν_3_ = 0 K). The dominant progression is the ν_1_ symmetric stretch populating O_3_ vibrational states from ν_1_ = 0 to ν_1_ = 5 with the minor progression being combination bands of ν_2_ = 1 with ν_1_ = 0 to ν_1_ = 5. The temperature of ν_3_ was found to have no significant contribution to the spectra, consistent with previous assignments (Arnold et al., 1994), and was therefore left at 0 K. The 0 K simulation provides an acceptable match to the photoelectron spectrum under optimal cold conditions.

**Table 1:** Vibrational energies used in Franck-Condon simulation (Arnold et al., 1994).

	**ν_1_ (100) (eV)**	**ν_2_ (010) (eV)**	**ν_3_ (001) (eV)**
${\text{O}}_{3}^{-}$$\tilde{X}$ ^2^B _1_	0.121	0.068	0.109
O_3_ $\tilde{X}$ ^1^A_1_	0.137	0.087	0.129

Overall COAT demonstrates the ability to thermalize the ions to a temperature cooler than through the use of supersonic expansion alone. This is consistent with similar accumulation traps that have been demonstrated to be effective at cooling and thermalizing both small (Hock et al., 2012) and large molecules (Boyarkin and Kopysov, 2014). As also found by other research groups (Wang and Wang, 2008; Hock et al., 2012), the degree of cooling is dependent on the buffer gas with the mixture of He/H_2_ being found to be more effective than neat He. The difference is observed as the suppression of pathway (2) and the significant reduction of pathway (3).

### Heating

Upon loading into COAT, precursor anions collide with the buffer gas transforming translational kinetic energy into rovibrational heating of the ions. Total photoelectron spectra under various heating conditions are shown in Figure 6 in contrast to the cooling conditions shown in Figure 5. These spectra are scaled to the 0–0 peak in the photodetachment channel (1) to more clearly distinguish the relative differences in dissociative events. Autodetachment peaks corresponding to electrons arising from ${\text{O}}_{2}^{-}$(v″ = 4, 5, and 6), with peaks located at 0.07, 0.19, and 0.32 eV respectively, are clearly resolved under all hot conditions. While v″ = 4 is observed under the conditions of no buffer gas (Figure 6), top panel, black trace), a significant enhancement in intensity of the ${\text{O}}_{2}^{-}$(v″ = 5,6) is observed under all hot COAT conditions (Figure 6). The sensitivity of the autodetachment channel to ${\text{O}}_{3}^{-}$ vibrational excitation infers a strong coupling of vibrational excitation within the ^2^A_2_ excited state to highly vibrationally excited products (Shen et al., 2017). The expansion of the parent anion wavefunction upon vibrational excitation of ${\text{O}}_{3}^{-}$ may lead to an increase in Franck-Condon overlap with the ^2^A_2_ excited state.

The ability to influence the dissociative pathways through heating the precursor ions is further demonstrated with channel (3). The 2-photon process is enhanced upon heating at similar laser power density. Under no buffer gas conditions (Figure 6), top panel, black trace) the amplitude for the O^−^ photodetachment is lower than under heating conditions (red and purple traces). This is consistent with increased FC overlap with the ^2^A_2_ state upon photoexcitation increasing the fraction of events from channels (2) and (3). The primary parameters affecting the heating of the ions are settings that affect the kinetic energy of the precursor anion, and trapping times. Settings such as Wiley-McLaren ion extraction voltage, the COAT float voltage, as well as the COAT entrance lens voltage can increase the temperatures of the ions by accelerating to larger translational energies just prior to initial collisions within COAT.

The hottest conditions (Figure 6), upper panel, red trace) were achieved with a combination of short trapping time (500 μs), He buffer gas, and accelerating ions with the entrance lens just before trapping. This resulted in a significantly larger contribution from the autodetachment channel (2) as well as the O^−^ photodissociation channel (3) with the 2-photon O^−^ signal amplitude dominating over the stable channel. Reducing the entrance lens acceleration voltage (by ~50 V) under the same source conditions (upper panel, purple line) still shows significant heating, but to a lesser degree as indicated by the amplitude of the autodetachment signal. The increased spectral congestion with the autodetachment channel is due to autodetachment to O_2_(v′ > 0), which has been previously reported in electron scattering experiments at high incident electron kinetic energy (Allan et al., 1996b). The dominant autodetachment channel produces O_2_(v′ = 0) + e^−^ even as the precursor ions are heated to a higher degree, as evidenced by the resolved ${\text{O}}_{2}^{-}$(v″ = 4,5,6) autodetachment peaks. This is in contrast to the DEA experiments carried out by Allan et al. where the distribution of autodetached electrons originate almost equally from the O_2_(v′ = 0) as from O_2_(v′ = 1) products as the impact electron kinetic energy increases. The difference may be a result of the internal energy distribution in collisionally activated precursor anions compared to the well-defined impact electron energy in the DEA experiments (Allan et al., 1996b). The increase in precursor ion temperatures does not appear to significantly increase the 2-photon signal from ${\text{O}}_{2}^{-}$(v″ < 4).

Under collimated laser conditions, the shortened trapping time of 800 μs in COAT (Figure 6), lower panel, brown trace) yields a larger distribution of autodetachment electrons as well as hot bands in contrast to the cold spectra (Figure 6), lower panel, blue trace). This indicates a higher average ion temperature than the ions thermalized for 30 ms at 300 K as shown in Figure 6 (bottom panel, black trace). Upon heating, the stable channel shows a significant increase in photodetachment hot bands, particularly for the $1_{1}^{0}$ and $1_{2}^{0}$ transitions. The amplitude for the hot bands in the bottom panel of Figure 6 is consistent with the expected trend from the amplitude of the autodetachment channel, with the shorter 800 μs trapping time exhibiting prominent peaks for $1_{1}^{0}$ and $1_{2}^{0}$ hot bands. In the hottest spectrum (Figure 6), top panel, red trace) it can be seen that the $1_{1}^{0}$ hot band is nearly half the amplitude of the 0–0 transition in that spectrum. Additionally, a significant increase in spectral congestion due to sequence bands is observed, most notably in the hottest spectrum where the $1_{0}^{2}$ peak exhibits the largest amplitude in the stable spectra.

A Franck-Condon simulation with the temperature of the vibrational modes set to ν_1_ = 2,000 K, ν_2_ = 1,500 K is shown in Figure 7, providing an estimate for the temperature of the ions under the hottest conditions observed. These temperatures should be considered a lower limit to the ion temperature given that a significant fraction of the ions undergo photodissociation, which is not taken into consideration in the simulation. The temperature difference in vibrational modes is explained by the expected non-Boltzmann distribution of vibrational excitation due to collisional heating as well as the opening of the photodissociation/autodetachment pathway with parent ion vibrational excitation. This excitation introduces competition between the stable and dissociative channels. Additionally, it has been found that the bending mode (ν_2_) significantly contributes to channel (2) (Shen et al., 2017). The sequence bands shown in the Franck-Condon simulation show that significant spectral congestion is caused by transitions from excitation of the ν_1_ and ν_2_ modes with up to three quanta of excitation in the precursor anion.

The heating of ions using COAT is shown to have a dependency on the voltage settings just prior to their entry into COAT, as well as the trapping duration. Increasing the kinetic energy of the ions prior to their entry into COAT and shorter trapping times resulted in hotter ions. In the case of ozonide, pathways (2) and (3) are significantly increased along with an increase in spectral congestion for pathway (1). This demonstrates an effective way to exert control over the ion temperature to examine the effects of internal excitation on dissociation dynamics and thermally activated processes.

## Conclusions

The addition of COAT to the PPC spectrometer enables the preparation of colder anions than achievable with a supersonic expansion alone, as well as the preparation of collisionally heated ions in a controlled manner. The elimination of the photodissociation/autodetachment channel (2) ${\text{O}}_{3}^{-}$ + hν → ${\text{O}}_{2}^{-}$(${}^{2}\Pi_{\text{g}}$, v > 4) + O(^3^P) channel demonstrates the ability for COAT to internally cool precursor anions. The enhancement of both channels (2) and (3) ${\text{O}}_{3}^{-}$ + hν → O^−^(^2^P) + O_2_(${}^{1}\Delta_{\text{g}}$), as well as the appearance of hot bands in the photoelectron spectrum for channel (1), indicate that varying trapping conditions can also be used to produce hot precursor anions. Most importantly, COAT has demonstrated the ability to influence the dissociation dynamics of ${\text{O}}_{3}^{-}$. Cooling precursor ions to their vibrational ground states will be invaluable in future experiments, including studies of much larger systems that have not been sufficiently cooled by supersonic expansion alone, such as the tert-butoxide anion (Shen et al., 2014). Well-characterized anion temperatures will also be integral for further laser excitation experiments, where the effects of direct infrared excitation of specific modes will be examined (Otto et al., 2014a,b; Ray et al., 2017). Of particular interest is cooling HOCO anions to their vibrational ground state prior to infrared photoexcitation. This will extend the work conducted on this system by studying how product branching ratios between OH + CO and H + CO_2_ as well as tunneling rates for HOCO → H + CO_2_ are impacted by controlled anion excitation (Lu et al., 2007; Johnson et al., 2014). Additionally, the ability to heat ions through collisional excitation can provide an approach for examination of thermally activated processes, providing increased flexibility in PPC spectroscopy.

## Data Availability

The raw data supporting the conclusions of this manuscript will be made available by the authors, without undue reservation, to any qualified researcher.

## Author Contributions

RC conceived the experiment. BS designed and assembled COAT. All authors assisted in modifying the PPC spectrometer to accommodate COAT and designed the experiments. BS, KL, and YB collected and analyzed the data. BS wrote the manuscript. KL, YB, and RC edited the manuscript.

### Conflict of Interest Statement

The authors declare that the research was conducted in the absence of any commercial or financial relationships that could be construed as a potential conflict of interest.

## References

[B1] Allan M. Asmis K. Popovic D. Stepanovic M. Mason N. Davies J. (1996a). Resonances in collisions of low-energy electrons with ozone: experimental elastic and vibrationally inelastic differential cross sections and dissociative attachment spectra. J. Phys. B Atom Mol Optic Phys. 29, 4727–4747. 10.1088/0953-4075/29/20/024

[B2] Allan M. Asmis K. Popovic D. Stepanovic M. Mason N. Davies J. (1996b). Production of vibrationally autodetaching O-2(-) in low-energy electron impact on ozone. J. Phys. B Atom. Mol. Optic. Phys. 29, 3487–3495. 10.1088/0953-4075/29/15/020

[B3] Arnold D. Xu C. Kim E. Neumark D. (1994). Study of low-lying electronic states of ozone by anion photoelectron-spectroscopy of ${\text{O}}_{3}^{-}$. J. Chem. Phys. 101, 912–922. 10.1063/1.467745

[B4] Bowen M. S. Continetti R. E. (2004). Photodetachment imaging study of the vinoxide anion. J. Phys. Chem. 108, 7827–7831. 10.1021/jp040083w

[B5] Boyarkin O. V. Kopysov V. (2014). Cryogenically cooled octupole ion trap for spectroscopy of biomolecular ions. Rev. Sci. Instru. 85:033105. 10.1063/1.4868178 24689562

[B6] Continetti R. (2001). Coincidence spectroscopy. Annu. Rev. Phys. Chem. 52, 165. 10.1146/annurev.physchem.52.1.165 11326063

[B7] Continetti R. E. (1998). Photoelectron-photofragment coincidence studies of dissociation dynamics. Int. Rev. Phys. Chem. 17, 227–260. 10.1080/014423598230144 31145616

[B8] Corderman R. Lineberger W. (1979). Negative-ion spectroscopy. Annu. Rev. Phys. Chem. 30, 347–378. 10.1146/annurev.pc.30.100179.002023

[B9] Cui Q. Morokuma K. (1998). Ab initio studies on the electronic excited states and photodissociation of O-3 anion. J. Chem. Phys. 108, 7684–7694. 10.1063/1.476322

[B10] Ervin K. Anusiewicz W. Skurski P. Simons J. Lineberger W. (2003). The only stable state of O-2(-) is the X (2)Pi(g) ground state and it (still!) has an adiabatic electron detachment energy of 0.45 eV. J. Phys. Chem. 107, 8521–8529. 10.1021/jp0357323

[B11] Ervin K. Ho J. Lineberger W. (1988). Ultraviolet photoelectron-spectrum of ${\text{NO}}_{2}^{-}$. J. Phys. Chem. 92, 5405–5412. 10.1021/j100330a017

[B12] Frisch M. J. Trucks G. W. Schlegel H. B. Scuseria G. E. Robb M. A. Cheeseman J. R. (2004). Gaussian 03, Revision B.04. Wallingford, CT: Gaussian, Inc.

[B13] Garner M. C. Hanold K. A. Resat M. S. Continetti R. E. (1997). Stability and dissociation dynamics of the low-lying excited states of ozone. J. Phys. Chem. 101, 6577–6582. 10.1021/jp9703519

[B14] Gerlich D. (1992). Inhomogeneous RF-fields - a versatile tool for the study of processes with slow ions. Adv. Chem. Phys. 82, 1–176. 10.1002/9780470141397.ch1

[B15] Gerlich D. (1995). Ion-neutral collisions in a 22-pole trap at very-low energies. Phys. Scripta T59, 256–263. 10.1088/0031-8949/1995/T59/035

[B16] Gerlich D. Jerke G. Muck U. Person U. (2019). Schnelles Ventil zur Erzeugung Sehr Kurzer Gasimpulse. Available online at: http://www.tu-chemnitz.de/physik/ION/Technology (accessed Febraury 27, 2017).

[B17] Goebbert D. Sanov A. (2009). Photodetachment, photofragmentation, and fragment autodetachment of [O_2_*n*(H_2_O)_*m*_]^−^ clusters: Core-anion structures and fragment energy partitioning. J. Chem. Phys. 131:104308 10.1063/1.3224135

[B18] Hock C. Kim J. B. Weichman M. L. Yacovitch T. I. Neumark D. M. (2012). Slow photoelectron velocity-map imaging spectroscopy of cold negative ions. J. Chem. Phys. 137:244201. 10.1063/1.4772406 23277929

[B19] Jasik J. Zabka J. Roithova J. Gerlich D. (2013). Infrared spectroscopy of trapped molecular dications below 4 K. Int. J. Mass Spectr. 354, 204–210. 10.1016/j.ijms.2013.06.007

[B20] Johnson C. Otto R. Continetti R. (2014). Spectroscopy and dynamics of the HOCO radical: insights into the OH + CO -> H + CO_2_ reaction. Phys. Chem. Chem. Phys. 16, 19091–19105. 10.1039/C4CP02593H 25098907

[B21] Johnson C. J. Shen B. B. Poad B. L. Continetti R. E. (2011). Photoelectron-photofragment coincidence spectroscopy in a cryogenically cooled linear electrostatic ion beam trap. Rev. Sci. Instru. 82:105105. 10.1063/1.3641875 22047327

[B22] Jones R. Anderson S. (2000). Simplified radio-frequency generator for driving ion guides, traps, and other capacitive loads. Rev. Sci. Instru. 71, 4335–4337. 10.1063/1.1318914

[B23] Jones R. Gerlich D. Anderson S. (1997). Simple radio-frequency power source for ion guides and ion traps. Rev. Sci. Instru. 68, 3357–3362. 10.1063/1.1148297

[B24] Kamrath M. Z. Garand E. Jordan P. A. Leavitt C. M. Wolk A. B. Van Stipdonk M. J.. (2011). Vibrational characterization of simple peptides using cryogenic infrared photodissociation of H-2-tagged, mass-selected ions. J. Am. Chem. Soc. 133, 6440–6448. 10.1021/ja200849g 21449591 PMC3099397

[B25] Liang J. Zheng H. Zhang X. Li R. Cui Z. (2007). Franck-Condon simulation of photoelectron spectroscopy of O-3: including duschinsky effects. J. Mol. Struct. Theochem. 814, 99–103. 10.1016/j.theochem.2007.03.002

[B26] Lu Z. Hu Q. Oakman J. E. Continetti R. E. (2007). Dynamics on the HOCO potential energy surface studied by dissociative photodetachment of HOCO– and DOCO–. J. Chem. Phys. 126:194305. 10.1063/1.2731787 17523802

[B27] Mann J. Troyer M. Jarrold C. (2015). Photoelectron imaging and photodissociation of ozonide in O-3(-)center dot(O-2)(n) (n = 1-4) clusters. J. Chem. Phys. 142 10.1063/1.4916048 25833577

[B28] Matejcik S. Kiendler A. Stampfli P. Stamatovic A. Märk T. D. (1996). Vibrationally resolved electron attachment to oxygen clusters. Phys. Rev. Lett. 77, 3771–3774. 10.1103/PhysRevLett.77.3771 10062304

[B29] Moriwaki Y. Tachikawa M. Maeno Y. Shimizu T. (1992). Collision cooling of ions stored in quadrupole radiofrequncy trap. Japn. J. Appli. Phys. 31, L1640–L1643. 10.1143/JJAP.31.L1640

[B30] Moriwaki Y. Tachikawa M. Shimizu T. (1996). Dependence of temperature of collision-cooled ions stored in an RF trap on trapping parameters. Japn. J. Appli. Phys. 35, 757–760. 10.1143/JJAP.35.757

[B31] Nestmann B. Kumar S. Peyerimhoff S. (2005). Contribution of Feshbach resonance to the 1.3-eV dissociative-electron-attachment cross section of ozone. Phys. Rev. 71:012705 10.1103/PhysRevA.71.012705

[B32] Novick S. Engelking P. Jones P. Futrell J. Lineberger W. C. (1979). Laser photoelectron, photodetachment, and photodestruction spectra of ${\text{O}}_{3}^{-}$. J. Chem. Phys. 70, 2652–2662. 10.1063/1.437842

[B33] Otto R. Ma J. Ray A. W. Daluz J. S. Li J. Guo H.. (2014a). Imaging dynamics on the F + H2O -> HF + OH potential energy surfaces from wells to barriers. Science 343, 396–399. 10.1126/science.1247424 24407479

[B34] Otto R. Ray A. W. Daluz J. S. Continetti R. E. (2014b). Direct IR excitation in a fast ion beam: application to NO-photodetachment cross sections. EPJ Tech. Instru. 1;3 10.1140/epjti3

[B35] Otto R. Xie J. Brox J. Trippel S. Stei M. Best T.. (2012). Reaction dynamics of temperature-variable anion water clusters studied with crossed beams and by direct dynamics. Faraday Discussions. 157, 41–57. 10.1039/c2fd20013a 23230763

[B36] Rangwala S. Kumar S. Krishnakumar E. Mason N. (1999). Cross sections for the dissociative electron attachment to ozone. J. Phys. B Atom. Mol. Optic. Phys. 32, 3795–3804. 10.1088/0953-4075/32/15/311

[B37] Ray A. W. Ma J. Otto R. Li J. Guo H. Continetti R. E. (2017). Effects of vibrational excitation on the F + H2O [rightward arrow] HF + OH reaction: dissociative photodetachment of overtone-excited [F-H-OH]. Chem. Sci. 8, 7821–7833. 10.1039/C7SC03364H 29163919 PMC5674243

[B38] Redwine J. Davis Z. Burke N. Oglesbee R. McLuckey S. Zwier T. (2013). A novel ion trap based tandem mass spectrometer for the spectroscopic study of cold gas phase polyatomic ions. Int. J. Mass Spectr. 348, 9–14. 10.1016/j.ijms.2013.04.002

[B39] Sanz M. E. McCarthy M. C. Thaddeus P. (2005). Vibrational excitation and relaxation of five polyatomic molecules in an electrical discharge. J. Chem. Phys. 122:194319. 10.1063/1.1869988 16161585

[B40] Shen B. Benitez Y. Lunny K. Continetti R. (2017). Internal energy dependence of the photodissociation dynamics of O-3(-) using cryogenic photoelectron-photofragment coincidence spectroscopy. J. Chem. Phys. 147:094307 10.1063/1.4986500 28886639

[B41] Shen B. Poad B. Continetti R. (2014). Photoelectron-photofragment coincidence studies of the tert-butoxide anion (CH3)(3)CO-, the carbanion isomer (CH3)(2)CH2COH-, and corresponding radicals. J. Phys. Chem. 118, 10223–10232. 10.1021/jp5090235 25289788

[B42] Smalley R. Wharton L. Levy D. (1977). Molecular optical spectroscopy with supersonic beams and jets. Accounts Chem. Res. 10, 139–145. 10.1021/ar50112a006

[B43] Stert V. Radloff W. Schulz C. Hertel I. (1999). Ultrafast photoelectron spectroscopy: Femtosecond pump-probe coincidence detection of ammonia cluster ions and electrons. Eur. Phys. J. 5, 97–106. 10.1007/s100530050234

[B44] Wang X. B. Wang L. S. (2008). Development of a low-temperature photoelectron spectroscopy instrument using an electrospray ion source and a cryogenically controlled ion trap. Rev. Sci. Instru. 79:073108. 10.1063/1.2957610 18681692

[B45] Wester R. (2009). Radiofrequency multipole traps: tools for spectroscopy and dynamics of cold molecular ions. J. Phys. B Atom. Mol. Optic. Phys. 42 10.1088/0953-4075/42/15/154001

[B46] Wiley W. McLaren I. (1955). Time-of-flight mass spectrometer with improved resolution. Rev. Sci. Instru. 26, 1150–1157. 10.1063/1.1715212

